# A long-term cross-sectional study with modified forgotten joint score to assess the perception of artificial joint after total knee arthroplasty

**DOI:** 10.1051/sicotj/2021013

**Published:** 2021-03-11

**Authors:** Karthik Chithartha, Anjaly S. Nair, Jai Thilak

**Affiliations:** 1 Resident in Orthopaedics, Amrita Institute of Medical Sciences Kochi 682041 Kerala India; 2 Amrita Institute of Medical Sciences Kochi 682041 Kerala India; 3 Clinical Professor in Orthopaedics, Amrita Institute of Medical Sciences Kochi 682041 Kerala India

**Keywords:** Total knee arthroplasty, Functional score, Perception of joint, Long term, PROMs, Forgotten joint score

## Abstract

*Background and purpose*: The ultimate goal for an arthroplasty surgeon is to provide the patient a joint that feels more like a natural joint. The Modified Forgotten Joint Score (MFJS) is a newly introduced functional scoring system that has a superior ability to assess this property among arthroplasty patients. The objective of this study is to evaluate the long-term temporal association of the MFJS and total knee arthroplasty (TKA). *Methods*: We assessed 360 patients post TKA with MFJS questionnaire. The patient groups were distributed at follow-up intervals of 3 weeks (*n* = 55), 6 months (*n* = 45), 1 year (*n* = 57), 2 years (*n* = 40), 3 years (*n* = 49), 5 years (*n* = 49), 7 years (*n* = 39), and 10 years (*n* = 26). Higher score suggests a forgotten artificial joint. *Results*: Post-operative mean MFJS scores were 64.4 ± 7.6 at 3 weeks, 87.7 ± 5.6 at 6 months, 89.2 ± 3.1 at 1 year, 89.9 ± 2.6 at 2 years, 89.4 ± 3.2 at 3 years, 89.1 ± 4 at 5 years, 84.5 ± 8.8 at 7 years, and 82.7 ± 11.9 at 10 years. The score at 3 weeks was significantly lesser than the average scores at other follow-up intervals. The score at 6 months was significantly higher compared to the score at 10 years. The average score at 1 year, 2 years, 3 years, and 5 years were significantly higher compared to the average score at 7 years and 10 years. *Conclusion*: The trend of the MFJS score was found to drastically improve from 3 weeks to 6 months and peak in 2 years after which the score tends to attain a plateau up to 5 years following which there is a decline in the score at 7- and 10-years post-surgery. Age did not have an influence on the variation in functional score in any of the follow-up groups. MFJS has a strong positive correlation with the well-recognised KOOS scoring system.

*Level of evidence*: IV

## Introduction

Among the joint problems, osteoarthritis (OA) is the most common and most frequent joint disease with a prevalence of 22–39% [1]. For patients with advanced knee OA, TKA may be the only option for pain relief and to improve function. The extremely high economic burden of osteoarthritis is largely attributed to the disability caused by arthritis, expense of treatment, and comorbid disease due to the change of lifestyle adapted by the patients [2]. However, while the surgeries for treating OA as such are costly, they also appear to be cost-effective in the long term [2]. Total knee arthroplasty (TKA) is the most widely practiced surgical option for arthritis all over the world. According to a survey 1,83,384 primary joint replacement surgeries were performed in the year 2018 [3].

TKA is a high-volume, high-cost medical intervention, hence numerous health-related quality-of-life outcome measures were developed to aid investigators to quantify improvements in TKA patient health status. Patient-reported data have been preferred to that of physician-based. Surgeon-based questionnaires and functional assessment of the surgery tend to give an outcome that is more than the actual perception of the knee by the patient. Harris et al. [4] in their study reported a disparity between patient and surgeon satisfaction after total knee arthroplasty. 94.5% of surgeons and 90.3% of patients recorded satisfaction after 12 months post-TKA. Swedish Knee Arthroplasty Registry has shown that 17% of patients were dissatisfied post-TKA as reported by Dunbar et al. [5]. Hence patient reported outcome measures to give the actual perception of the joint by the patient and consequently the outcome of TKA. There is a clinically important difference between joint perception and satisfaction. The ultimate goal in joint replacement is when the patient “forgets” the artificial joint i.e., feel like a natural joint. The forgotten joint score (FJS-12) has a superior ability to detect a forgotten joint [6]. FJS-12 has been widely studied and found to be superior to other scoring systems in terms of internal consistency, low ceiling effect, and responsiveness [6–11]. Modifications have been done for the original FJS-12 and the modified forgotten joint score is used in this study as it has a lower percentage of missing data and a low ceiling effect [12].

The primary objective of the study is to detect the mean MFJS score of patient groups belonging to each follow-up interval and assess the trend or variation in the score over a period of 10 years. The hypothesis is that the MFJS score shows a dramatic improvement after a period following the surgery after which the score peaks and remains plateau and starts to decline after a period of time. The only study available in the literature that has validated the newly introduced MFJS scoring system is the study by Lavery et al. [12]. Hence in this study, the newly introduced MFJS questionnaire is correlated with the widely accepted and validated Knee Injury and Osteoarthritis Outcome Score (KOOS) scoring system [13–16].

## Methods

### Study design

In total 1200 patients who had undergone TKA in the same institution from January 2008 to November 2018 were screened from the surgery registry after obtaining approval from the ethical committee. After screening patients with the exclusion criteria: Age < 40 years, any previous surgery in the knee, revision TKA, unicondylar and bicompartmental knee replacements, American Society of Anaesthesiologists physical status score (ASA) > 3, inflammatory arthritis, patients who were operated on by other surgeons were excluded and only TKA performed by the same single surgeon were included in the study to remove the surgeon-based bias.

At defined postoperative intervals, the patients were either phoned to complete the MFJS and KOOS questionnaire, or the questionnaire was filled in person during follow-up. Scores were attained for the patient subgroups of 3 weeks, 6 months, 1 year, 2 years, 3 years, 5 years, 7 years, and 10 years. Among the 430 eligible patients who were called 90 patients were not reachable through phone or postal, hence a total of 360 patients were included in the study. No single patient was included in multiple follow-up intervals.

An orthopaedic surgeon with 15 years of experience in knee arthroplasty performed the surgeries. The same surgical technique was carried out throughout the study interval. The surgical approach was the same for all patients with median parapatellar arthrotomy and femur and tibia was prepared with appropriate cutting jigs and trial components. Either Cruciate retaining or PCL substituting implants were used. The patella was resurfaced for some patients. Most of the subjects had undergone conventional TKA, however, two other advanced methods of arthroplasty such as navigation TKAs and robotic TKAs were also performed for some patients. Routine postoperative care and physiotherapy protocol was initiated for all patients (Table 1).

**Table 1:** Patient demographics.

	3 weeks	6 months	1 year	2 years	3 years	5 years	7 years	10 years
	(*n* = 55)	(*n* = 45)	(*n* = 57)	(*n* = 40)	(*n* = 49)	(*n* = 49)	(*n* = 39)	(*n* = 26)
AGE	64.64 ± 7.7	67.40 ± 7.7	68.25 ± 6.5	67.82 ± 7.0	69.92 ± 6.9	70.3 ± 6.6	72.4 ± 7.3	77.4 ± 8.9
Sex male	13 (23.6%)	10 (22.2%)	9 (15.7%)	5 (12.5%)	6 (12.2%)	6 (12.2%)	8 (20.5%)	5 (19.2%)
ASA								
1	6	7	4	7	7	9	3	4
2	46	34	52	29	37	37	32	24
3	3	4	1	4	5	3	4	2
Number of unilateral TKAs	31 (56.3%)	20 (44.4%)	21 (36.8%)	13 (32.5%)	20 (40.8%)	18 (36.7%)	14 (35.8%)	13 (50%)
Number of Cruciate Retaining TKAs	31 (56.3%)	27 (60%)	23 (40.3%)	9 (22.5%)	15 (30.6%)	23 (46.9%)	10 (25.6%)	1 (3.8%)
Number of Patella resurfaced TKAs	38 (69%)	34 (75.5%)	24 (42.1%)	7 (17.5%)	10 (20.4%)	29 (59.1%)	22 (56.4%)	21 (80.7%)

### Statistical analysis

Statistical analysis was performed using IBM SPSS version 20.0 software [17]. Categorical variables were expressed using frequency and percentage. With 90% power and 95% confidence the minimal sample size required in each time slot was calculated and the overall minimal sample size required was found to be 256 samples. Numerical variables were presented using mean and standard deviation. ANOVA test was used to compare age and MFJS score between different follow-up intervals and for significance, Bonferroni multiple comparison tests was applied for pairwise comparison. Pearson’s correlation coefficient between MFJS and KOOS score was computed and its significance was tested using linear reg *t* test. A *P*-value of <0.05 was considered to be statistically significant.

## Results

**Figure 1:**
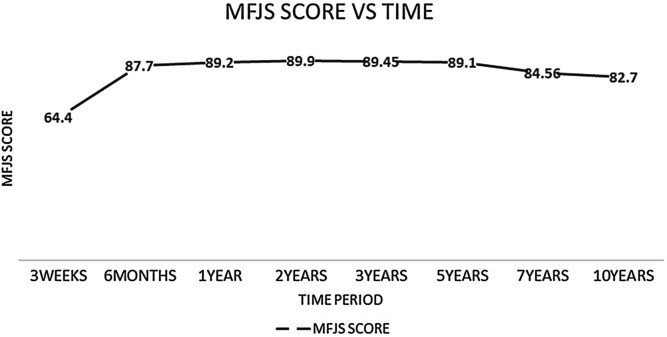
The trend of the MFJS score at each follow-up interval.

In total 360 patients were involved in the study. The post-operative MFJS score averages were least at 3 weeks and highest at 2 years. The average score at 3 weeks was significantly lesser than the average score at all other follow up intervals (*p* < 0.001). The average score at 10 years was significantly lesser than the average score at 6 months (*p* = 0.044), 1 year (*p* = 0.001), 2 years (*p* < 0.001), 3 years (*p* = 0.001), 5 years (*p* = 0.001) post-operative. The MFJS score at 7 years was significantly lesser than the average score at 1 year (*p* = 0.006), 2 years (*p* = 0.003), 3 years (*p* = 0.005), 5 years (*p* = 0.010) post-operative (Figure 1).

**Figure 2:**
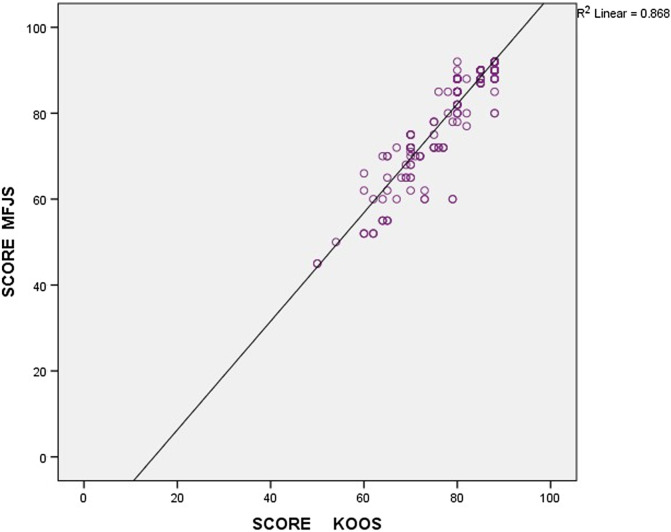
Pearson correlation between MFJS and KOOS.

There was a strong positive correlation between the MFJS and the KOOS score (Pearson Correlation 0.931) (Figure 2).

## Discussion

The study of the pattern of improvement in functional outcome post-TKA has been attempted by only a handful of studies and the literature provides contradicting statements regarding the same. Most previous studies [18–20] have certain limitations that only clinician based outcome scores or general health survey scores were used, rather than using patient-reported outcomes measures (PROMs), which are of actual importance to the patients, or the time intervals at which the patients were assessed were too spaced out, thereby missing the true timeline. Our study uses PROMs for the assessment of patients at closely spaced intervals.

No study in the literature to the best of our knowledge has analysed the MFJS score over a long-term follow-up interval in post-TKA patients. This might be the first study to analyse the MFJS score over a 10 year follow-up period. According to this study, patients who have undergone TKA showed significant improvement in the MFJS score between 3 weeks and 6 months post-surgery. However, no significant difference was detected between 6 months and 1–5 years postoperative. The score begins to decline by 7 years and a significant difference in the score was noticed between 1 and 5 years post-operative to that of 7 years and 10 years postoperative interval.

A study by Hiyama et al. [21] gives a similar comparison to the current analysis in terms of short-term results. In this study, a drastic increase in the FJS score was noted between 1 month and 6 months postoperative. No significant difference was noted between 6 months and 1-year postoperative interval. Another study by Carlson et al. [22] also shows drastic improvement in FJS immediately after surgery and reaching a plateau from 1 to 3 years followed by a drop in the score at 5 years. The current analysis with MFJS also shows significant improvement between 3 weeks and 6 months postoperative interval and a plateau is reached by 6 months to 5 years. Furthermore, the prolonged follow-up period analysis in the current study shows that the mean MFJS declines significantly in 7 and 10 years post-operative intervals.

The MFJS score is said to be excellent if the score is in the range of 87.5–100% [12]. In the current study, the score reaches excellent from 6th month onwards and stays the same till 5 years. Another study conducted by Williams et al. [23] also gives a similar conclusion, where the OKS score was used and the score peaked at 2 years and plateaued up to 4 years following which the score had a declining trend through to 10 years.

**Figure 3:**
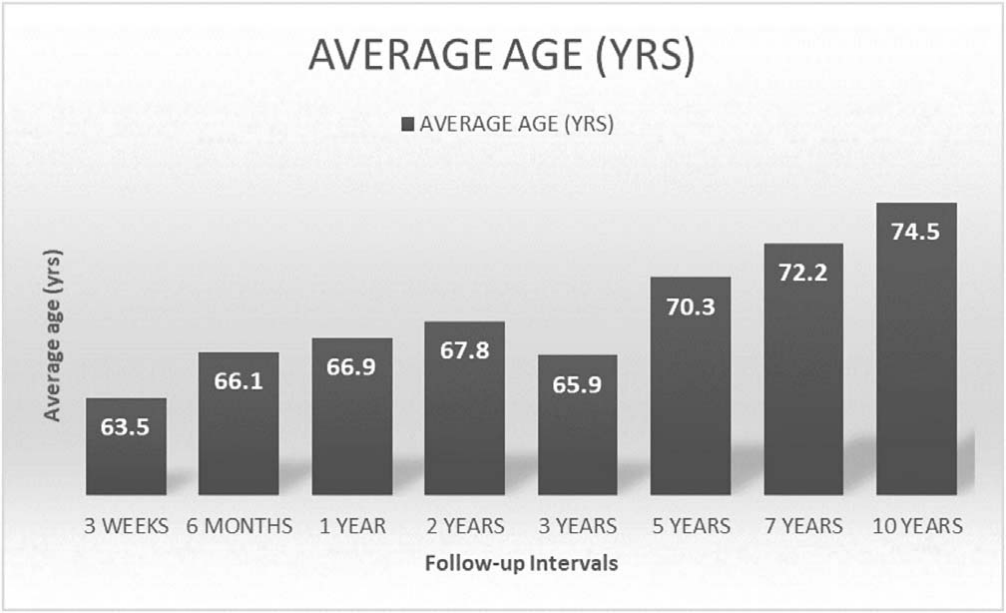
Average age of the study group at each follow-up interval.

A recent prospective study was conducted by Tiwari et al. [24] where the temporal pattern of clinical outcome scores in TKA patients for a period of 5 years post-operative was assessed. WOMAC, American Knee Society score, SF-36 scores were obtained. Improvement in the score from 6 months to 2 years was observed among various functional scores analysed. There was a variable decline in functional score especially in patients aged >68 years, whereas the score plateaued after 2 years in patients aged <68 years. The age of the study population in the 7- and 10-year follow-up interval was high, 72.2 and 74.5 respectively (Figure 3) in the current analysis. Hence, to rule out the influence of age as the reason for the decline of the functional score in these groups, the MFJS score of patients belonging to >70 years of age in the 7- and 10-year follow-up group was compared with that of patients belonging to >70 years of age in the rest of the follow-up groups (1–5 year). The Mean MFJS score of patients belonging to the 7- and 10-year follow-up group was found to be statistically lower (*P*-value 0.002) compared to the other group. Therefore, higher age in the 7- and 10-year follow-up group has not influenced the variation in functional score in these groups contradicting the findings by Tiwari et al. [24].

### Strengths and limitations

To the best of our knowledge, this is one among the very few studies to make use of the simple and more recently introduced MFJS score on TKA patients. We have collected data on long-term follow-up patients, up to 10 years post-surgery. Closely spaced intervals between the follow-up groups help us determine the variations in score accurately. All patients operated by a single surgeon using the standard technique in the same institute. Validation of the MFJS score with that of the widely studied and accepted KOOS scoring system.

The limitations of the study being it is a cross-sectional study. A prospective long-term follow-up study of the same patient group with pre-operative baseline other PROMs score would have been ideal. However, due to feasibility issues, this was not possible. Other confounding variables such as BMI at the time of surgery could have been included in the study.

## Conclusion

This study assessed the trend of the MFJS score post Total Knee Arthroplasty and found the score to drastically improve from 3 weeks to 6 months and peak in 2 years after which the score tends to attain a plateau up to 5 years following which there is a decline in the score at 7- and 10-years post-surgery. In addition, we found that age did not have an influence on the variation in functional score in any of the follow-up groups. MFJS has a strong positive correlation with the well-recognised KOOS scoring system and can be routinely used to assess the postoperative outcome in TKA patients.

## Conflict of interest

KC certifies that he has no financial conflict of interest in connection with this article.

AN certifies that she has no financial conflict of interest in connection with this article.

JT certifies that he has no financial conflict of interest in connection with this article.

## References

[1] Pal CP, Singh P, Chaturvedi S, Pruthi KK, Vij A (2016) Epidemiology of knee osteoarthritis in India and related factors. Indian J Orthop 50, 518–522. 27746495 10.4103/0019-5413.189608 PMC5017174

[2] Bitton R (2009) The economic burden of osteoarthritis. Am J Manag Care 15, S230–S235. 19817509

[3] Vaidya SV, Jogani AD, Pachore JA, Armstrong R, Vaidya CS (2020) India joining the world of hip and knee registries: Present status – a leap forward. Indian J Orthop 55, 1–10. 10.1007/s43465-020-00251-y PMC8149501 34122754

[4] Harris IA, Harris AM, Naylor JM, Adie S, Mittal R, Dao AT (2013) Discordance between patient and surgeon satisfaction after total joint arthroplasty. J Arthroplasty 28, 722–727. 23462496 10.1016/j.arth.2012.07.044

[5] Dunbar MJ, Richardson G, Robertsson O (2013) I can’t get no satisfaction after my total knee replacement: Rhymes and reasons. Bone Joint J 95-B, 148–152. 24187375 10.1302/0301-620X.95B11.32767

[6] Heijbel S, Naili JE, Hedin A, W-Dahl A, Nilsson KG, Hedström M (2020) The forgotten joint score-12 in Swedish patients undergoing knee arthroplasty: A validation study with the Knee Injury and Osteoarthritis Outcome Score (KOOS) as comparator. Acta Orthop 91, 88–93. 31711349 10.1080/17453674.2019.1689327 PMC7006730

[7] Wang Z, Deng W, Shao H, Zhou Y, Li H (2020) Forgotten joint score thresholds for forgotten joint status and patient satisfaction after unicompartmental knee arthroplasty in Chinese patients. J Arthroplasty 35(10), 2825–2829. 32482475 10.1016/j.arth.2020.05.010

[8] Behrend H, Giesinger K, Giesinger JM, Kuster MS (2012) The “forgotten joint” as the ultimate goal in joint arthroplasty: Validation of a new patient-reported outcome measure. J Arthroplasty 27, 430–436.e1. 22000572 10.1016/j.arth.2011.06.035

[9] Giesinger K, Hamilton DF, Jost B, Holzner B, Giesinger JM (2014) Comparative responsiveness of outcome measures for total knee arthroplasty. Osteoarthr Cartil 22, 184–189. 10.1016/j.joca.2013.11.001 PMC3988962 24262431

[10] Azzi E, Thienpont E, Avaux M, Houssiau FA, Durez P (2014) The forgotten joint score, a new questionnaire to evaluate patient’s perception of total knee and hip arthroplasty in patients with established rheumatoid arthritis. Ann Rheum Dis 73, 1173.

[11] Thomsen MG, Latifi R, Kallemose T, Barfod KW, Husted H, Troelsen A (2016) Good validity and reliability of the forgotten joint score in evaluating the outcome of total knee arthroplasty: A retrospective cross-sectional survey-based study. Acta Orthop 87, 280–285. 26937689 10.3109/17453674.2016.1156934 PMC4900097

[12] Lavery J, Anthony I, Blyth M, Jones B (2018) The validity and reliability of the modified forgotten joint score. J Orthop 15(2), 480–485. 29881181 10.1016/j.jor.2018.03.029 PMC5990363

[13] Gandek B, Ware JEJ (2017) Validity and responsiveness of the Knee Injury and Osteoarthritis Outcome Score: A comparative study among total knee replacement patients. Arthritis Care Res (Hoboken) 69, 817–825. 28085998 10.1002/acr.23193 PMC5449223

[14] Peer MA, Lane J (2013) The Knee Injury and Osteoarthritis Outcome Score (KOOS): A review of its psychometric properties in people undergoing total knee arthroplasty. J Orthop Sports Phys Ther 43, 20–28. 23221356 10.2519/jospt.2013.4057

[15] Collins NJ, Prinsen CAC, Christensen R, Bartels EM, Terwee CB, Roos EM (2016) Knee Injury and Osteoarthritis Outcome Score (KOOS): Systematic review and meta-analysis of measurement properties. Osteoarthr Cartil 24, 1317–1329. 10.1016/j.joca.2016.03.010 27012756

[16] Roos EM, Toksvig-Larsen S (2003) Knee injury and Osteoarthritis Outcome Score (KOOS) – validation and comparison to the WOMAC in total knee replacement. Health Qual Life Outcomes 1, 17. 12801417 10.1186/1477-7525-1-17 PMC161802

[17] Anon (2011) IBM SPSS Statistics for Windows.

[18] Noble PC, Scuderi GR, Brekke AC, Sikorskii A, Benjamin JB, Lonner JH, Chadha P, Daylamani DA, Scott WN, Bourne RB (2012) Development of a new knee society scoring system. Clin Orthop Relat Res 470, 20–32. 22065240 10.1007/s11999-011-2152-z PMC3237986

[19] Webster K, Feller J (2015) Comparison of the short form-12 (SF-12) health status questionnaire with the SF-36 in patients with knee osteoarthritis who have replacement surgery knee surgery. Sport Traumatol Arthrosc 24, 2620–2626. 10.1007/s00167-015-3904-1 26821809

[20] Teo BJX, Koh JSB, Jiang L, Allen JC, Yeo SJ, Sen HT (2019) Association of the 36-Item short form health survey physical component summary score with patient satisfaction and improvement 2 years after total knee arthroplasty. JAMA Netw Open 2(2), e190062. 30794301 10.1001/jamanetworkopen.2019.0062 PMC6484598

[21] Hiyama Y, Wada O, Nakakita S, Mizuno K (2016) Joint awareness after total knee arthroplasty is affected by pain and quadriceps strength. Orthop Traumatol Surg Res 102, 435–439. 27052936 10.1016/j.otsr.2016.02.007

[22] Carlson VR, Post ZD, Orozco FR, Davis DM, Lutz RW, Ong AC (2018) When does the knee feel normal again: A cross-sectional study assessing the forgotten joint score in patients after total knee arthroplasty. J Arthroplasty 33, 700–703. 29108793 10.1016/j.arth.2017.09.063

[23] Williams DP, Blakey CM, Hadfield SG, Murray DW, Price AJ, Field RE (2013) Long-term trends in the Oxford knee score following total knee replacement. Bone Joint J 95-B, 45–51. 23307672 10.1302/0301-620X.95B1.28573

[24] Tiwari V, Lee J, Sharma G, Kang YG, Kim TK (2019) Temporal patterns of commonly used clinical outcome scales during a 5-year period after total knee arthroplasty. J Orthop Traumatol 20, 16. 30911852 10.1186/s10195-019-0520-8 PMC6434008

